# P-1734. Pharmacist-Led Antibiotic Deprescribing for Negative Urine Cultures in the Emergency Department

**DOI:** 10.1093/ofid/ofae631.1898

**Published:** 2025-01-29

**Authors:** Ashley Teter, Evan Hurley

**Affiliations:** Inova Health System, Alexandria, Virginia; Inova Health System, Alexandria, Virginia

## Abstract

**Background:**

Inappropriate antibiotic use in EDs, often due to empiric prescribing before urine culture results are available, poses significant challenges. This study evaluates the impact of a pharmacist-led intervention to deprescribe antibiotics for patients with negative urine culture results.

Intervention

**Figure:**
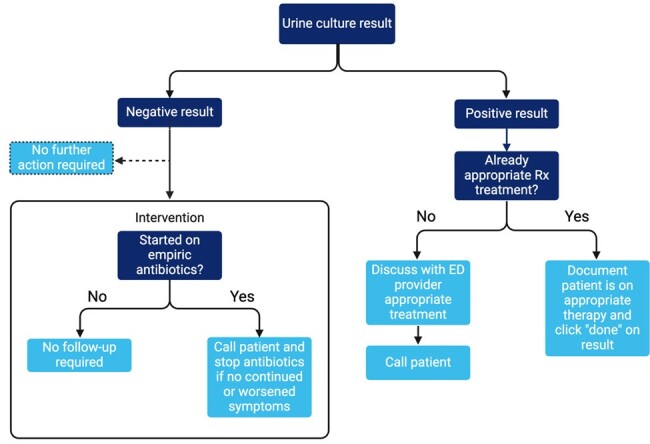

**Methods:**

Conducted from May 1, 2023, to March 31, 2024, this study employed a two-phase approach involving a retrospective review and a prospective intervention. Inclusion criteria were patients prescribed antibiotics for a UTI with subsequent negative urine culture results during an ED visit. Exclusion criteria included patients admitted for inpatient treatment or transferred to other healthcare facilities. In Phase I (May to July 2023), a clinical pharmacist in the ED adjusted antibiotic therapy based on culture susceptibility results on weekdays. Phase II (January to March 2024) expanded to include antibiotic deprescribing in patients with negative results. The primary outcome was the number of ED readmissions within 30 days after antibiotic discontinuation for UTI symptoms or serious infection. Secondary outcomes included total antibiotic days saved, antibiotic days prescribed per patient at ED discharge, and post-intervention antibiotic days per patient. Statistical analyses utilized the Fisher Exact Test and Mann-Whitney U Test.

Patient Characteristics

**Figure:**
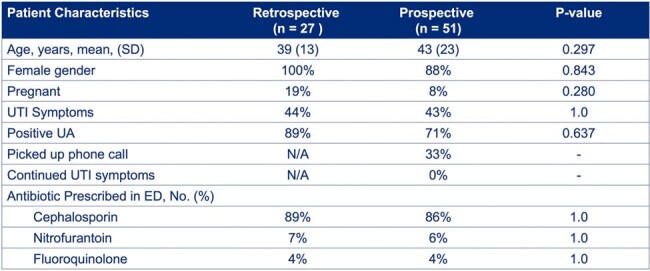

**Results:**

The study included 78 patients: 27 in the retrospective phase and 51 in the prospective phase. Among the initial 51 patients, follow-ups with 17 revealed no continued UTI symptoms. There were no readmissions in our prospective intervention group compared to two in the retrospective group (P=0.117). Antibiotic days were significantly reduced from a pre-intervention average of 7.2 to 5.2 days per patient post-intervention (P=0.000468), saving a total of 82 antibiotic days.

Results

**Figure:**
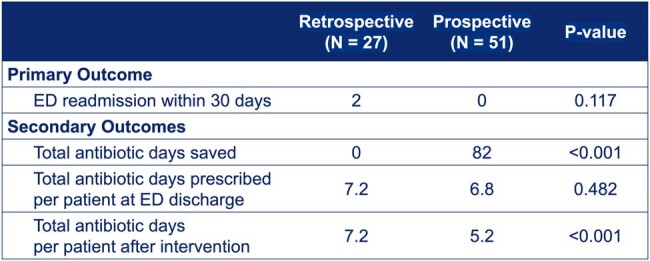

**Conclusion:**

Pharmacist-led deprescribing of antibiotics in patients with negative urine cultures significantly reduced total antibiotic days per patient without increasing ED readmissions for urinary tract infection symptoms. These findings demonstrate the feasibility and safety of deprescribing in a clinical ED setting and suggest potential changes in clinical practice, advocating for the expansion of pharmacist roles in antibiotic stewardship programs.

Results Continued

**Figure:**
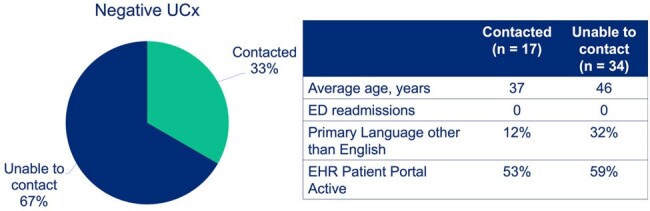

**Disclosures:**

**All Authors**: No reported disclosures

